# Autopsy findings of previously described case of diffuse intrinsic pontine glioma-like tumor with EZHIP expression and molecular features of PFA ependymoma

**DOI:** 10.1186/s40478-021-01215-5

**Published:** 2021-06-23

**Authors:** Murad Alturkustani, Jennifer A. Cotter, Roshan Mahabir, Debra Hawes, Carl Koschmann, Sriram Venneti, Alexander R. Judkins, Linda J. Szymanski

**Affiliations:** 1 Keck School of Medicine, University of Southern California, Los Angeles, CA USA; 2 Department of Pathology and Laboratory Medicine, Children’s Hospital of Los Angeles, Los Angeles, CA USA; 3 Department of Pathology, University of Michigan School of Medicine, Ann Arbor, MI USA; 4 Division of Pediatric Hematology/Oncology, Department of Pediatrics, C.S. Mott Children’s Hospital, University of Michigan, 1500 E Medical Center Dr, Ann Arbor, MI 48109 USA; 5 Laboratory of Brain Tumor Metabolism and Epigenetics, Department of Pathology, University of Michigan, 3520E MSRB 1, 1150 W. Medical Center, Ann Arbor, MI 48109 USA; 6 King Abdulaziz University, Jeddah, Saudi Arabia

We wanted to expand on the pathological findings of the recently published case report of a diffuse intrinsic pontine glioma (DIPG)-like tumor with EZHIP expression and methylation features of childhood posterior fossa group A (PFA) ependymoma by Pratt et al. [5] by describing the postmortem brain examination findings. The previous case report described an unusual brainstem glioma with characteristic radiographic and histopathologic features of DIPG (based on biopsy), but with methylation features of PFA ependymoma. The 5-year-old male patient underwent multiple rounds of radiation, but died 18 months later. At the family’s request, a postmortem examination was performed to better characterize this tumor.

**Fig. 1:**
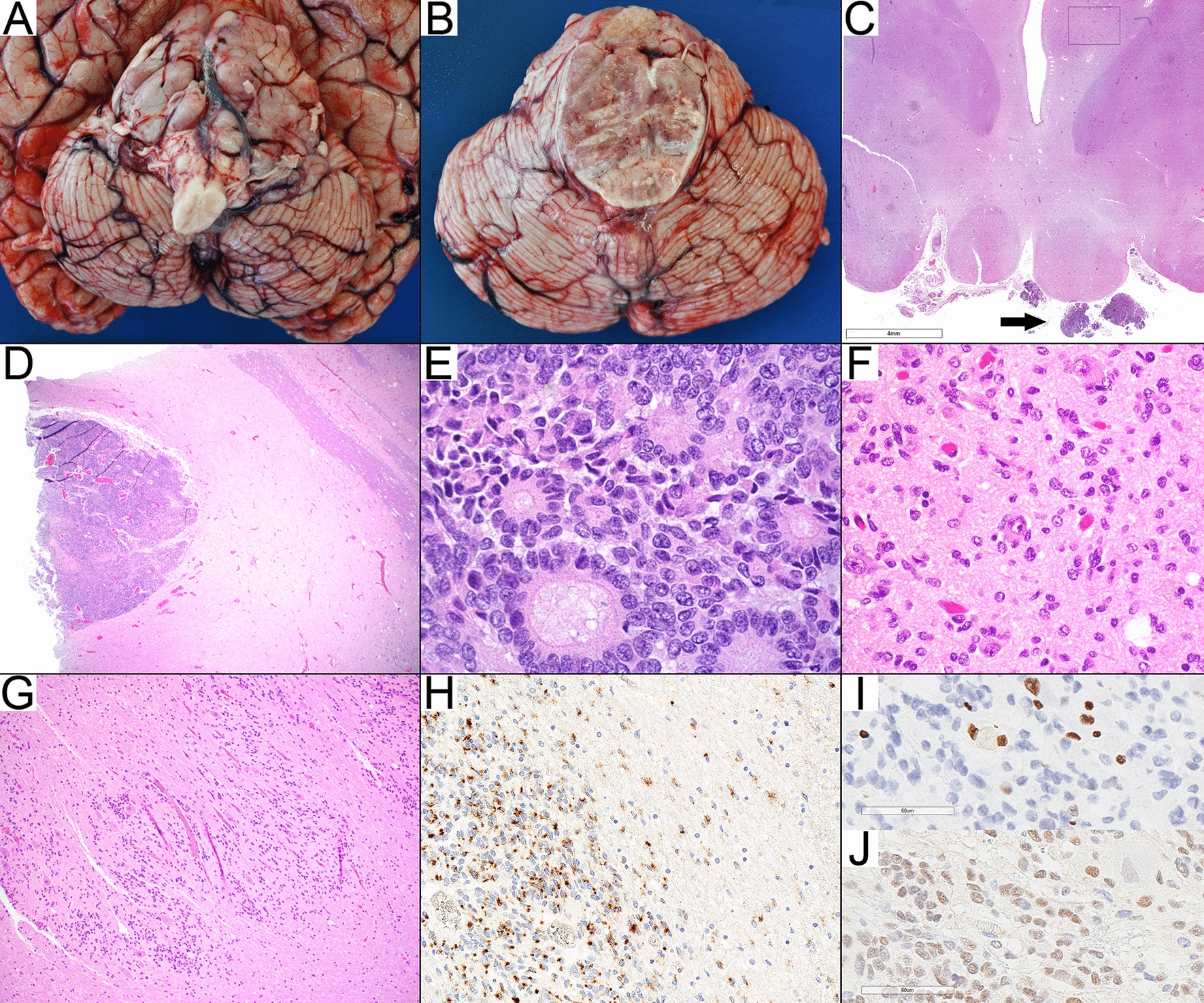
Autopsy findings. **a** Lobulated brainstem tumor. **b** Pons with solid and infiltrative tumor growth. **c**. Leptomeningeal spread (arrow) at the level of mamillary bodies and infiltrative neoplastic cells in the ventral thalamus as indicated by the square (see **f**). **d**, **e**. Solid well-circumscribed nodule of ependymal differentiation with true ependymal rosettes in the pons (H&E, D:40× , E:600×). **f**. Infiltrative tumor involving the thalamus, H&E, 400× . **g** Infiltrative tumor involving the cerebellar dentate nucleus, H&E, 100× . **h** EMA staining in solid and infiltrative components, 200× . **i**. H3K27me3 staining is lost in the infiltrative neoplastic cells while retained in the entrapped neurons and endothelial cells, 400× .**j**. EZHIP (CXorf67) staining highlights the infiltrative component while negative in the endothelial cells and the entrapped neurons 400×

Postmortem examination of the brain confirmed that the brainstem was expanded by a large lobulated tan-gray tumor that distorted the cranial nerves and encased the basilar artery (Fig. 1a, b). There was opacification of the leptomeninges near the mammillary bodies corresponding to leptomeningeal spread (Fig. 1c). Axial sections through the brainstem revealed the tumor was partially solid and infiltrative, centered in the pons and extended into the midbrain and cerebellum. Solid tumor nodules were noted near the fourth ventricle.

Histopathological examination of the pons demonstrated a tumor with two distinct growth patterns consisting of either solid nodules of hypercellular tumor or diffusely infiltrative tumor. Neoplastic cells in both components demonstrated eosinophilic cytoplasm, and oval nuclei with a salt-and-pepper chromatin pattern. In the solid component within the pons, ependymal rosettes, perivascular pseudorosettes (Fig. 1d, e) and focal areas of astroblastic rosettes occasionally surrounding hyalinized blood vessels suggestive of ependymal differentiation were encountered. Minimal necrotic changes were noted that were likely secondary to treatment related effects. There was no significant endothelial proliferation or mitotic activity.

The infiltrative component extended beyond the pons and involved the midbrain, bilateral thalami (Fig. 1f), left basal ganglia and temporal lobe superiorly; the medulla inferiorly, and the cerebellar white matter, cortex and dentate nuclei (Fig. 1g) dorsally as well as the basal leptomeninges and leptomeninges of the mamillary bodies. Throughout, tumor cells formed Scherer’s secondary structures around neurons and blood vessels, under the pia and along white matter tracts. There were rare mitoses. The tumor cells were viable and there were no significant treatment related effects. The immunohistochemical findings were similar in both components. Immunohistochemistry showed tumor immunoreactivity for GFAP, EMA (strong and diffuse dot-like positivity) (Fig. 1h), and EZHIP (Fig. 1j). There was loss of nuclear expression of H3K27me3 (Fig. 1i).

Local infiltration can be seen in low-grade glial neoplasms including ependymomas. However, an infiltrative growth pattern is typically considered diagnostic of a diffuse glioma and is not the expected behavior in an ependymoma. Distal infiltration is a criterion used by the recent cIMPACT-NOW guidelines to distinguish diffuse midline glioma from other well-circumscribed gliomas with H3K27 mutation [4]. This tumor demonstrates a fairly unique histology with a prominent infiltrative pattern of growth which we have not previously encountered nor is well described in ependymomas.

This case illustrates the challenges in incorporating the morphologic and molecular features of some pediatric tumors into a single integrated diagnosis based on the current WHO classification of tumors of the central nervous system [3]. The histology within the solid components and immunohistochemical profile of GFAP positivity and EMA perinuclear dot-like positivity is consistent with a classic ependymoma without high-grade features. Olig2 was not interpretable in the autopsy specimen as internal positive control was negative, but Olig2 was reported as negative in the preceding biopsy sample. While this lack of expression is more consistent with ependymal differentiation, it is not 100% specific as a subset of astrocytomas lack Olig2 expression as well [2]. The absence of nuclear expression of H3K27me3 and expression of EZHIP by immunohistochemical staining further helps in subclassification of the tumor as a PFA ependymoma. At the same time, the histology within the infiltrative pattern of growth with rare mitoses is consistent with a diffuse glioma without high-grade features (brisk mitotic count, microvascular proliferation or necrosis). In that context, the same pattern of H3K27me3 and EZHIP expression has been recently described in a subclass of diffuse midline glioma and would therefore establish a different diagnosis [1]. Consequently, this tumor poses a challenge for neuropathologists as the diagnosis rendered may vary based both on where the tumor was biopsied (solid vs infiltrative nodules) and their interpretation of the current WHO classification and cIMPACT-NOW guidelines.

In summary, this unusual brain tumor arising within the pons clinically met the criteria for a DIPG but upon biopsy examination demonstrated H3K27me3 global reduction, EZHIP overexpression and methylation profile that cluster with group ‘EPN, PFA’ despite a lack of apparent ependymal histologic features [5]. Subsequent postmortem examination revealed both well-developed ependymal features and extensive infiltrative growth and leptomeningeal spread, not detected clinically or radiologically. This difference highlights the importance of autopsy examination in characterizing tumor histology and growth pattern to better understand the full range of features of these complex diseases. This case also illustrates the difficulties that may be encountered in harmonizing the distinct molecular and histopathological features of some pediatric CNS tumors within the current WHO classification and cIMPACT-NOW guidelines. This will perhaps be aided by further study and characterization of the infiltrative component of this lesion, but also perhaps by the continued development of pediatric specific CNS entities within the WHO classification and cIMPACT-NOW guidelines.
